# A species-specific triplex PCR assay for authentication of *Galli Gigerii Endothelium Corneum*

**DOI:** 10.1080/23802359.2021.1899075

**Published:** 2021-03-19

**Authors:** Yuli Zhang, Juan Li, Shiqing Yang, Chao Xu, Roselyn Tehzee Gblinwon, Jianhui Hu, Xiaoxiang Sun, Guohua Xia, Huan Yang, Yuping Shen

**Affiliations:** a School of Pharmacy, Jiangsu University, Zhenjiang, China; b Department of Pharmacy, Zhenjiang Hospital of Traditional Chinese Medicine, Zhenjiang, China

**Keywords:** *Galli Gigerii Endothelium Corneum*, triplex PCR, authentication

## Abstract

A triplex PCR assay was developed to identify animal species and adulteration of a natural medicine *Galli Gigerii Endothelium Corneum* (GGEC). Three species-specific primer sets were designed according to the difference in mitochondrial genome of *Gallus gallus domesticus*, *Anas platyrhynchos* and *Anser anse*. The PCR conditions were optimized and the assay was well validated for high specificity and sensitivity (1 mg/μL). Especially, when artificial adulterants made from the mixture of three species were analyzed, the assay has still exhibited strong capability of differentiation. By using this developed method, two batches out of fourteen commercial GGEC products were identified to be adulterated by *Anser anse*. The newly proposed assay showed sufficient merits as a regular tool for the identification of counterfeits or adulterants of GGEC product for their pulverized and processed form, and even Chinese patent medicines composed of these species.

## 1. Introduction

*Galli Gigerii Endothelium Corneum* (GGEC), is the dry stomach inner-wall of *Gallus gallus domesticus* (GD). This well-known animal-derived natural medicine has been widely used in Traditional Chinese Medicine (TCM) clinics for more than 2000 years. GGEC has significant effect on dyspepsia and popularly consumed by child patients without side-effects observed. In addition, GGEC is one of major ingredients not only in 155 Chinese patent medicines for the treatment of spermatorrhea, enuresis, gallstones and so on (Chinese Pharmacopoeia Committee 2020), but functional foods or health care products for general population. *Anas platyrhynchos* (AP) and *Anser anser* (AA) were both of non-medicinal effects, but they were often used to make GGEC adulterant or counterfeits. Therefore, accurate identification of their animal origins is a prerequisite task to ensure its efficacy (Izadpanah et al. 2018; Jiang et al. 2018; Wang et al. 2020), however similar morphological characteristics and lacking professional experience make it difficult to distinguish the species of closer phylogenetic relationship. Some technologies based on chromatography and mass spectrometry have been developed for the analysis of natural products, which require complicated procedure and expensive instrument (Yang et al. 2017; Lin et al. 2020). However, similar chemical properties always lead to a large difficulty in accurate identification of a mixture.

In recent decades, polymerase chain reaction (PCR) shows a great advantage in convenience, specificity, and sensitivity for species identification (Girish et al. 2005; Chen et al. 2011, 2019a; Xu et al. 2015; Yang et al. 2019, 2020; Zheng et al. 2019; Zia et al. 2020). Especially, multiplex PCR incorporating species-specific amplification was much more preferred to accomplish this purpose, which offers reliable analysis of various species simultaneously in a mixed DNA template without expensive equipment and special reagents (Kitpipit et al. 2014; Palavesam et al. 2018; Prusakova et al. 2018; Chen et al. 2019b; Jiao et al. 2020). In this study, a triplex PCR assay was newly established to authenticate GGEC after validation for specificity and sensitivity.

## 2. Material and methods

### 2.1. Samples

**Figure 1.**
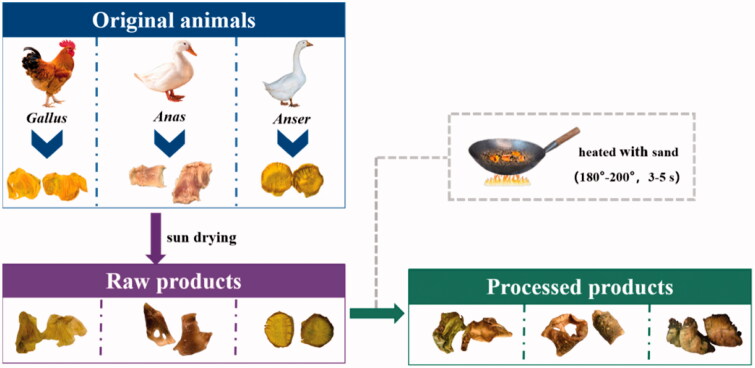
Procedures for self-made raw materials and processed products.

Fifteen batches of dry stomach inner-wall from *Gallus gallus domesticus*, *Anas platyrhynchos* and *Anser anser* were collected from market in various cities of China in 2019, and were coded GD1–GD5, AP1–AP5 and AA1–AA5 (Table 1). Their specimens were deposited at herbarium (Dr. Huan Yang, yanghuan1980@ujs.edu.cn) located in School of Pharmacy, Jiangsu University, PRC. All these raw materials were subjected to COI barcoding (Chinese Pharmacopoeia Committee 2020) or reported characteristic primer (Miguel et al. 2003; Chen et al. 2019a) for species verification immediately after collection (data shown in Supplementary Material). As illustrated in Figure 1, they were then processed in accordance with the protocols recorded in the prevailing Chinese Pharmacopoeia. Furthermore, fourteen batches of commercial products including raw GGEC (G1–G5) and processed GGEC (G6–G14) were purchased from different manufacturers. All the above collected samples were pulverized to be fine powder and stored in an electronic desiccator (*RH* < 35%) at room temperature prior to any further experiments.

**Table 1.** Raw materials used in the study.

Code	Species	Sources	Collection date	Voucher No.
GD1	*Gallus gallus domesticus*	Suzhou, Anhui, PRC	Jul, 2019	2019GD01
GD2	*Gallus gallus domesticus*	Zhenjang, Jiangsu, PRC	Sep, 2019	2019GD02
GD3	*Gallus gallus domesticus*	Zhenjang, Jiangsu, PRC	Sep, 2019	2019GD03
GD4	*Gallus gallus domesticus*	Huangshan, Anhui, PRC	Oct, 2019	2019GD04
GD5	*Gallus gallus domesticus*	Anqing, Anhui, PRC	Oct, 2019	2019GD05
AP1	*Anas platyrhynchos*	Zhenjang, Jiangsu, PRC	Sep, 2019	2019AP01
AP2	*Anas platyrhynchos*	Zhenjang, Jiangsu, PRC	Feb, 2019	2019AP02
AP3	*Anas platyrhynchos*	Jiangyin, Jiangsu, PRC	Jul, 2019	2019AP03
AP4	*Anas platyrhynchos*	Jiangyin, Jiangsu, PRC	Jul, 2019	2019AP04
AP5	*Anas platyrhynchos*	Jiangyin, Jiangsu, PRC	Jul, 2019	2019AP05
AA1	*Anser anser*	Zhenjang, Jiangsu, PRC	Mar, 2019	2019AA01
AA2	*Anser anser*	Zhenjang, Jiangsu, PRC	Aug, 2019	2019AA02
AA3	*Anser anser*	Taizhou, Jiangsu, PRC	Feb, 2019	2019AA03
AA4	*Anser anser*	Taizhou, Jiangsu, PRC	Jul, 2019	2019AA04
AA5	*Anser anser*	Taizhou, Jiangsu, PRC	Aug, 2019	2019AA05

### 2.2. DNA extraction

DNA of the samples was extracted and purified by SDS-based method according to a published paper (Yang et al. 2018). In detail, 50 mg of the homogenized sample was mixed with 995 μL of extraction buffer composed of 100 m*M* NaCl, 10 m*M* Tris-HCl (pH 8.0), 25 m*M* EDTA, 0.5%(*w/v*) SDS and 5 μL proteinase K (20 mg/mL), and the mixture was incubated at 56 °C for 6 h. For purification of DNA template, an equal volume of Tris-phenol solution, phenol-chloroform-isopentanol (PCI) solution and chloroform-isopentanol (CI) solution were mixed sequentially with the supernatants after centrifugation of the mixture at 12,000 rpm for 15 min in stages. Then, 450 μL of the supernatant was precipitated by 900 μL of 96% ethanol and 45 μL of 5.0 *M* KAc after constant incubation overnight at −20 °C. The supernatant was removed after centrifugation at 12,000 rpm for 15 min, and the resulting DNA pellet was washed with 70% ethanol and finally reconstituted in 25 μL of TE buffer (pH 8.0) for subsequent experiments. These DNA samples extracted from raw materials or processed products were diluted to 10 mg/μL, and those from highly processed products were used directly as template in further PCR assays. Then, the purity and concentration of all extracted DNA was measured using nucleic acid & protein spectrophotometer (Nano Drop 2000, Thermo, USA) based on absorbance at both A260/A280 and A260/A230.

### 2.3. Primer design

Species-specific primers were designed by Oligo software (v. 7.60, Molecular Biology Insights, Inc., Cascade, CO, USA) according to mitochondrial genome sequences of three species. The primers were subjected to evaluation by DNAMAN (v. 8.0.8.789, Lynnon Bio soft, San Ramon, CA, USA), then the assessed primers were synthesized by Sangon Biotech (Shanghai) Co., Ltd (China) and kept at −20 °C prior to subsequent PCR assays.

### 2.4. Pcr amplification

PCR was performed in a 25 μL reaction mixture containing 2.5 μL of 10 × PCR buffer, 2.5 μL of 2.0 m*M* MgCl_2_, 0.5 μL of 0.2 m*M* dNTPs, 0.5 μL of each primer set, 0.625 unit of Taq polymerase, 1 μL of 10 mg DNA template and distilled water (filled to a final volume of 25 μL). The optimization of primer concentration was carried out in the range of 0.12 μ*M*–0.28 μ*M.*

After assessment of annealing-temperature, all PCR assays were performed on a Bio-Rad T100 Thermal Cycler with an initial denaturation at 95 °C for 3 min, followed by 35 cycles of 95 °C for 30 s, 57 °C for 30 s and 72 °C for 1 min with a final extension at 72 °C for 5 min. The resulting PCR amplicons were visualized in 2% agarose gel electrophoresis stained with Ethidium bromide under UV illumination.

### 2.5. Validation of multiplex PCR assay

The developed multiplex PCR method was validated for specificity and sensitivity. The specificity test was performed by amplification of DNA extracted from different batches of processed GD, AP, and AA samples, respectively. Then, the sensitivity was evaluated on premixed DNA templates of each target species at four concentrations ranging from 0.01 mg/μL to 10 mg/μL.

### 2.6. Analysis of artificial adulterated samples

Those three species (*Gallus gallus domesticus*, *Anas platyrhynchos* and *Anser anser*) were mixed in seven proportions (3:2:1, 2:1:3, 1:3:2, 1:1:1, 2:3:1, 3:1:2, and 1:2:3) to make artificial adulterants, and the weight of each mixture was 50 mg. DNA of them was extracted, and after that, these DNA templates were amplified using the developed triplex PCR assay.

### 2.7. Authentication of commercial products

Finally, fourteen batches of commercial products including raw GGEC (G1–G5) and processed GGEC (G6–G14) purchased from different manufacturers were analyzed for the identification of *Anas platyrhynchos* and *Anser anse*, and the verification of labeling compliance by the established multiplex PCR assay.

## 3. Results and discussion

### 3.1. Triplex PCR conditions

**Figure 2.**
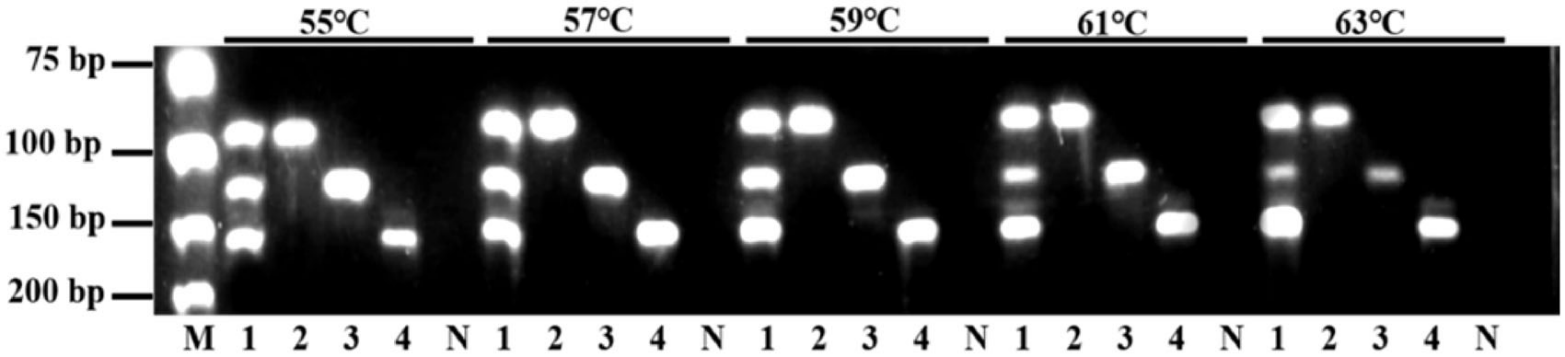
Optimization of annealing temperature for triplex PCR assay. Lane 1: GD: AP: AA (1:1:1); Lane 1: GD; Lane 1: AP; Lane 1: AA; M: DNA marker; N: Negative control.

Triplex PCR assay developed in this study aims for simultaneous detection of *Gallus gallus domesticus*, *Anas platyrhynchos*, and *Anser anse*. The primer sets designed for triplex PCR assay of three target species were showed in Table 2. Crucial optimization of annealing temperature (55 °C, 57 °C, 59 °C, 61 °C or 63 °C; Table 3) for triplex PCR conditions was illustrated in Figure 2. Three primer sets (0.20 μ*M* PGD, 0.20 μ*M* PAP and 0.20 μ*M* PAA) have well amplified the mixed DNA templates after 35 cycles while the best annealing temperature was 57 °C for elimination of nonspecific amplification.

**Table 2.** Designed species-specific primers.

Species	Code	Sequence (5’–3’)	Amplicon size	Accession No.	Gene Names	Target Range (nt)
		GCAGGTGACAGATTCTACTCC				
*Gallus gallus domesticus*	PGD	CTATTCAAGTGAAGCCTGGAC	94 bp	NC_001323.1	Nil	708–781
		ATGATTCCCCATACACGCAAA				
*Anas platyrhynchos*	PAP	CGGACTAGAATCCATTACCTG	124 bp	NC_009684.1	ND5	13,061–13,164
		CCATGTTACGAATAGGGCAAT				
*Anser anser*	PAA	TGACAATCCTCCTAACCCCAA	155 bp	NC_011196.1	ND5	11,893–12,027

**Table 3.** Experimental parameters for optimization of annealing temperature and primer concentration.

DNA templates	Optimization of annealing temperature		Optimization of primer concentration	
	Temperature	Other parameters		
GD: AP: AA (1:1:1) GD AP AA	55 °C	Primer concentration: 0.20 μM PGD, 0.20 μM PAP and 0.20 μM PAA Cycles: 35	Primer concentration	0.20 μ*M* PGA, 0.20 μ*M* PAP, and 0.20 μ*M* PAA
	57 °C			0.16 μ*M* PGA, 0.24 μ*M* PAP, and 0.16 μ*M* PAA
	59 °C			0.12 μ*M* PGA, 0.28 μ*M* PAP, and 0.12 μ*M* PAA
	61 °C			0.14 μ*M* PGA, 0.24 μ*M* PAP, and 0.14μ*M* PAA
	63 °C		Temperature	57 °C
			Cycles	35

**Figure 3.**
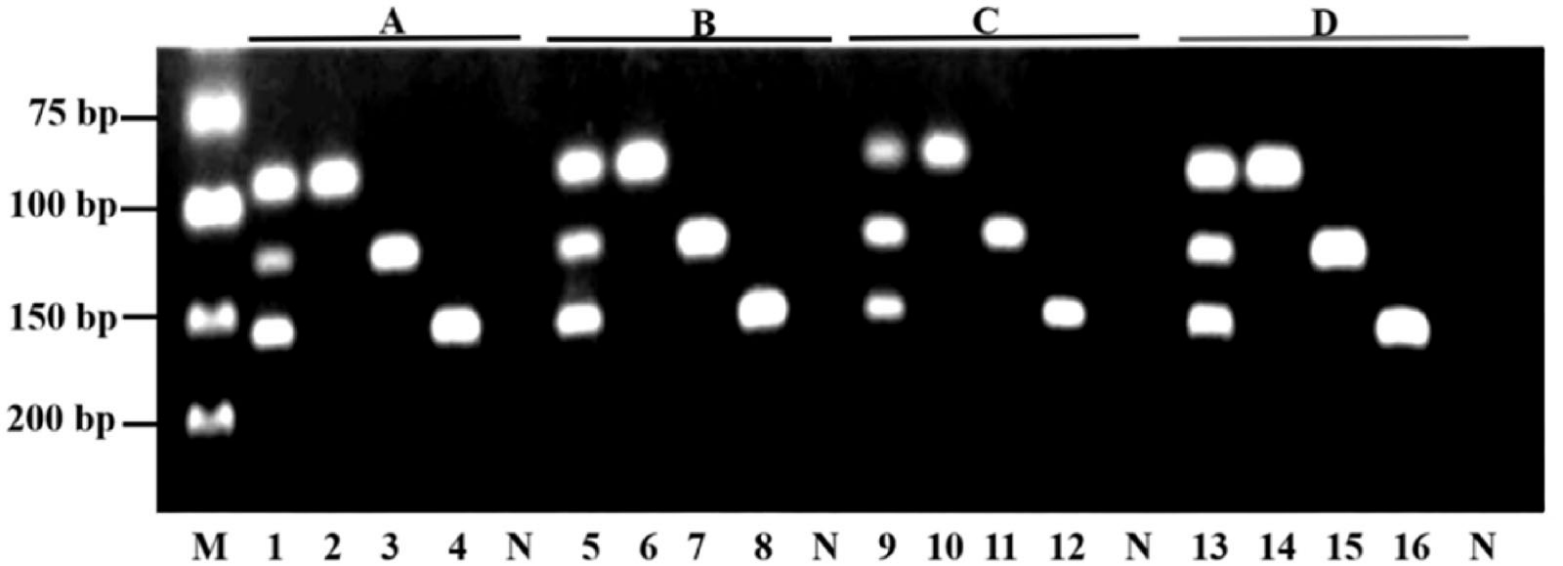
Optimization of primer concentration for triplex PCR assay. M: DNA marker; Lane 1–4: 0.20 μ*M* PGA, 0.20 μ*M* PAP, and 0.20 μ*M* PAA; Lane 5–8: 0.16 μ*M* PGA, 0.24 μ*M* PAP, and 0.16 μ*M* PAA; Lane 9–12: 0.12 μ*M* PGA, 0.28 μ*M* PAP, and 0.12 μ*M* PAA; Lane 13–16: 0.14 μ*M* PGA, 0.24 μ*M* PAP, and 0.14 μ*M* PAA; N: Negative control.

Three primer mixtures containing different final concentration of each primer set (Table 3) were examined in the optimization step. Figure 3 shows the agarose gel electrophoresis of amplicon resulted from triplex PCR assay using three different primer mixtures. Primer mixture containing 0.14 μ*M* PGD, 0.24 μ*M* PAP and 0.14 μ*M* PAA is chosen as the optimized primers concentration, evidenced by the consistently high band intensity at 94 bp, 124 bp and 155 bp for *Gallus gallus* domesticus, *Anas platyrhynchos* and *Anser anser*, respectively.

### 3.2. Specificity

**Figure 4.**
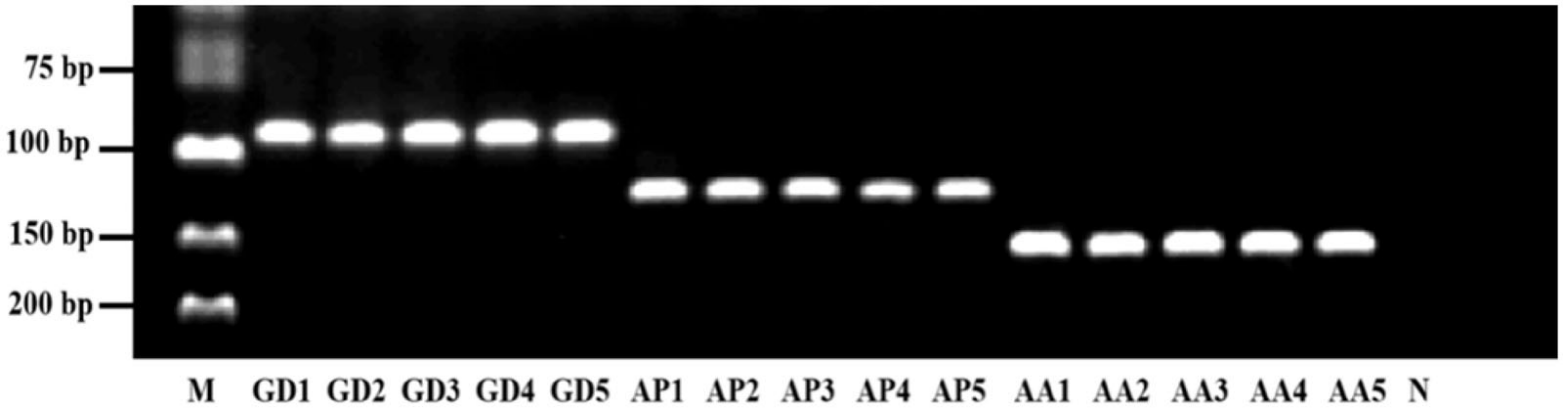
Specificity test. M: DNA marker; GD1–GD5: *Gallus gallus domesticus*; AP1–AP5: *Anas platyrhynchos*; AA1–AA5: *Anser anse*; N: negative control.

In this study, the specificity of this assay was evaluated by triplex PCR amplification against three individual DNA templates. As shown in Figure 4, clear individual bands were exhibited for their corresponding templates in agarose gel electrophoresis, demonstrating high specificity of the assay.

### 3.3. Sensitivity

**Figure 5.**
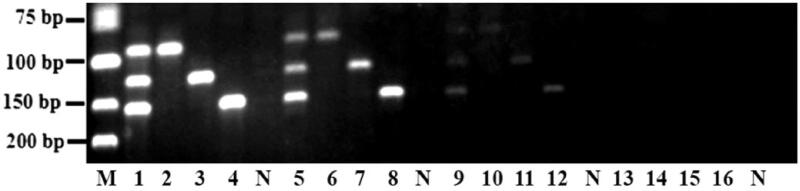
Sensitivity test. M: DNA marker; Lane 1–4: 10 ng/μL; Lane 5–8: 1 ng/μL; Lane 9–12: 0.1 ng/μL; Lane 13–16: 0.01 ng/μL; N: negative control.

To assess the sensitivity of the developed triplex PCR assay, serially diluted DNA of *Gallus gallus domesticus* was investigated. As shown in Figure 5, the band intensity gradually decreased as the template concentration was reduced from 10 mg/μL to 0.1 mg/μL. Then, very faint band was observed when using 0.1 mg/μl DNA template and no bands was observed when the template concentration was further decreased to 0.01 mg/μL. Taking into account that there could be day-by-day variation of the gel documentation system and agarose gel electrophoresis (technical variation), the detection limit of the triplex PCR assay was determined at 1 mg/μL for all three species.

### 3.4. Analysis of artificial adulterated samples

**Figure 6.**
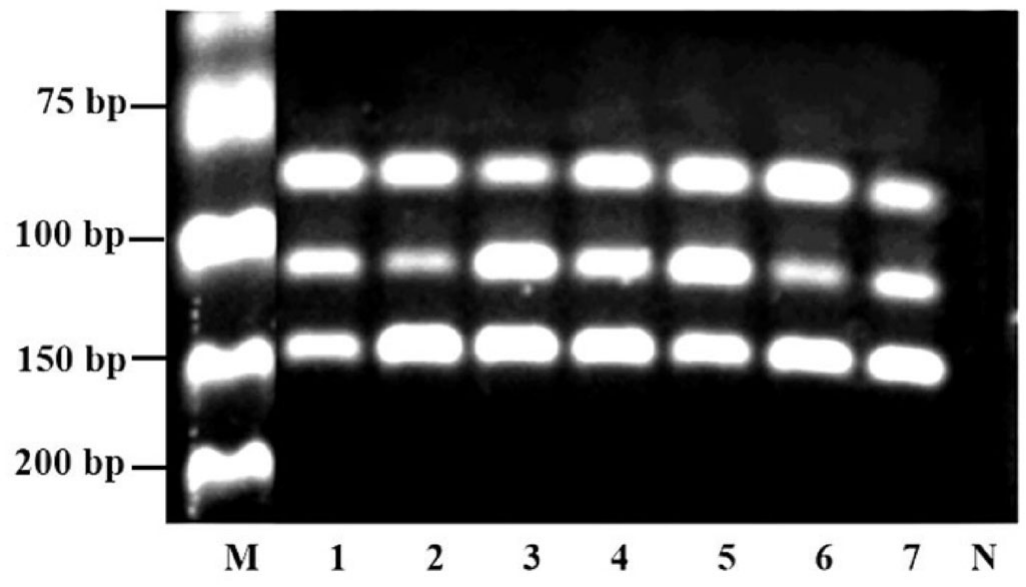
Analysis of artificial adulteration. M: DNA marker; Lane 1–7: Sample mixture (GD: AP: AA) in proportion of 3:2:1, 2:1:3, 1:3:2, 1:1:1, 2:3:1, 3:1:2, 1:2:3; N: negative control.

As shown in Figure 6, three corresponding species in seven artificial adulterants have been all detected by the triplex PCR assay. The band intensity is in accordance to proportional component in the mixture. And, the amplified bands were fairly clear even if the assay was applied to analyze samples, which demonstrated that the assay could be applied to the identification of *Anas platyrhynchos* and *Anser anser* in adulterated products.

### 3.5. Authentication of commercial products

**Figure 7.**
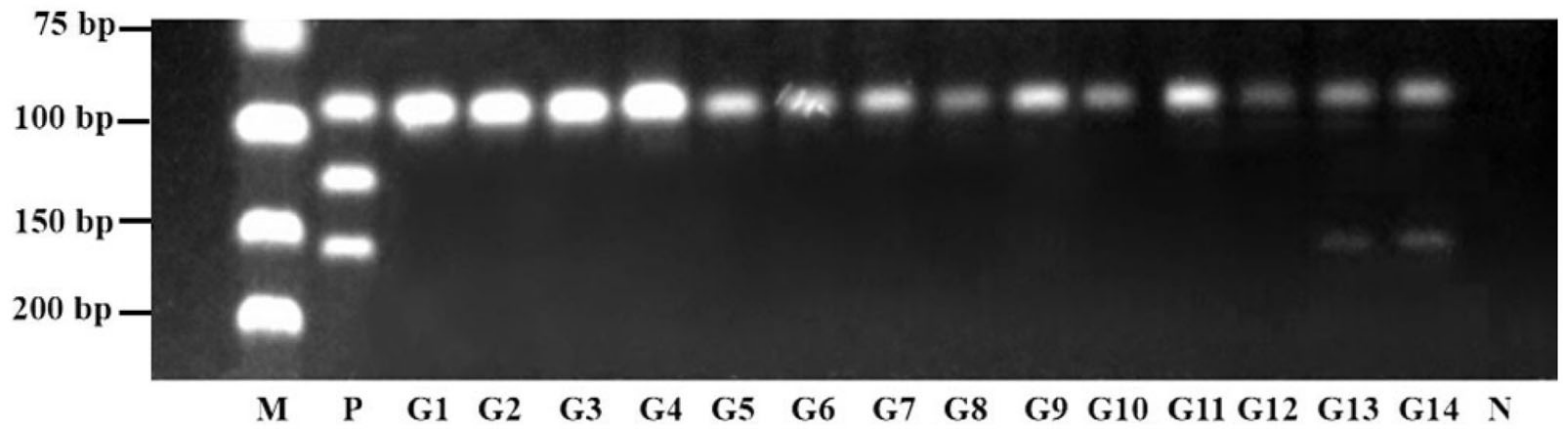
Authentication of commercial products by triplex PCR assay. M: DNA marker; P: Positive control; G1–G5: raw GGEC; G6–G14: processed GGEC; N: Negative control.

Fourteen batches of commercial products were subjected to the developed triplex PCR assay to identify *Anas platyrhynchos* and *Anser anser* that might adulterate the product. The results were shown in Figure 7 and summarized in Table 4. DNA template extracted from all these products even the processed GGEC have been successfully amplified. In previous studies, a single PCR assay was employed to distinguish the three species in a published report (Miguel et al. 2003), while the analyzed DNA was extracted from liver, muscle, fat, or binary mixture, however this method was not appropriate in processed stomach inner-wall (Pu et al. 2019). Due to DNA damage during processing possibly (Nor et al. 2021), the bands of raw GGEC (G1–G5) were brighter than of processed GGEC (G6–G14). And 12 out of the fourteen samples were authenticated as genuine GA product. However, it was also found that two batches (G13 and G14) were adulterated by *Anser anser.*

**Table 4.** Results of species identification in commercial GGEC products.

Code	Label	B/N	Manufacturers/sources	GD	AP	AA
G1	Raw GGEC	190213	Bozhou Yonggang Co., Ltd, Bozhou City, Anhui Province, PRC	+	−	−
G2	Raw GGEC	180809	Shandong Weifang Pharmaceutical Co., Ltd, Weifang City, Shandong Province, PRC	+	−	−
G3	Raw GGEC	200502	Bozhou Huifeng Guoyao Co., Ltd, Bozhou City, Anhui Province, PRC	+	−	−
G4	Raw GGEC	–	Xiaoxian Traditional Chinese Medicine Hospital, Suzhou City, Anhui Province, PRC	+	−	−
G5	Raw GGEC	19030101	Taizhou Baicao Co., Ltd, TaizhouCity,Jiangsu Province, PRC	+	−	−
G6	Processed GGEC	190316	Bozhou Yonggang Co., Ltd, Bozhou City, Anhui Province, PRC	+	−	−
G7	Processed GGEC	190501	Bozhou Yonggang Co., Ltd, Bozhou City, Anhui Province, PRC	+	−	−
G8	Processed GGEC	190523	Bozhou Yonggang Co., Ltd, Bozhou City, Anhui Province, PRC	+	−	−
G9	Processed GGEC	180716	Bozhou Yonggang Co., Ltd, BozhouCity, Anhui Province, PRC	+	−	−
G10	Processed GGEC	18110101	Taizhou Baicao Co., Ltd, Taizhou City, Jiangsu Province, PRC	+	−	−
G11	Processed GGEC	190213	Bozhou Yonggang Co., Ltd, Bozhou City, Anhui Province, PRC	+	−	−
G12	Processed GGEC	–	Nanjing Traditional Chinese Medicine Hospital, Nanjing City, Jiangsu Province, PRC	+	−	−
G13	Processed GGEC	190501	Jiangsu Jibeier Co., Ltd, Zhenjiang City, Jiangsu Province, PRC	+	−	+
G14	Processed GGEC	181001	Jiangsu Jibeier Co., Ltd, Zhenjiang City, Jiangsu Province, PRC	+	−	+

+: Positive; −: negative.

## 4. Conclusion

As a well-known natural medicine derived from *Gallus gallus domesticus*, GGEC is apt to be adulterated by stomach inner-wall of *Anas platyrhynchos* and *Anser anser*. In this study, a species-specific triplex PCR assay was newly established for simultaneous identification of GGEC products and two adulterants. After optimization of annealing-temperature and primer concentration, the developed assay exhibited a high specificity against target DNA fragments and the detection limit was determine to be 1 ng/μL of all three species. By this assay, *Anas platyrhynchos* and *Anser anser* were identified from artificial mixed samples, and two out of fourteen commercial products were identified to be adulterants mixed by *Anser anser*. Consequently, the proposed approach showed great merits as a routine method to authenticate GGEC in raw and processed forms.

## Data Availability

The sequence data that support the findings of this study are openly available in GenBank of NCBI at the website (https://www.ncbi.nlm.nih.gov/) under accession no. JQ627347 (*Gallus gallus*), MH744426 (*Anas platyrhynchos*), and MN122908 (*Anser anser*).
